# Quality of care in cystic fibrosis: assessment protocol of the French QIP PHARE-M

**DOI:** 10.1186/s13023-017-0749-3

**Published:** 2018-02-08

**Authors:** Dominique Pougheon Bertrand, Emmanuel Nowak, Clémence Dehillotte, Lydie Lemmonier, Gilles Rault

**Affiliations:** 1 LEPS EA 3412, Sorbonne Paris Cité, Bobigny, France; 2 INSERM CIC 1412 CHRU Brest, Brest, France; 3 Vaincre la Mucoviscidose, Paris, France; 4 CF Centre, Fondation ildys, Roscoff, France

**Keywords:** Cystic fibrosis, Quality improvement program, Quantitative study, Patient registry, Qualitative study

## Abstract

**Background:**

The PHARE-M care quality improvement program, modeled on the US Cystic Fibrosis Quality Improvement Program, was introduced at 14 cystic fibrosis centers (CFCs) in the French Cystic Fibrosis Network between 2011 and 2013. The pilot phase assessments attested the progressive adherence of the teams and improvements in care management. The PHARE-M Performance research project aims at assessing in 2015 the impact of the PHARE-M program on patient health indicators at trained versus untrained centers. It also sought to identify contextual factors that could account for variability in the performance of the PHARE-M among the trained centers.

**Methods:**

A mixed methodology combining:

- a quantitative experimental study: a comparison, using a mixed model for repeated data (from 2011 to 2015), of the average changes over time in forced expiratory volume in 1 s (FEV1) and body mass index (BMI) between two groups of patients included in a closed cohort (non-transplant patients, continuous follow-up at one participating CFC, and a CF-causing mutation), one having benefitted from the PHARE-M program and the other not having done so, and
- a realistic study: a characterization of the impact on care management and an identification of mechanisms through which the PHARE-M intervention improved the team’s effectiveness in different CFC contexts; this required modeling the intervention, context, and impact on care management with respect to the criteria of the chronic care model (CCM); this was done using a self-administered questionnaire given to professionals and patients/parents supplemented with focus groups.

**Conclusion:**

Although the study population was controlled, it may be difficult to establish a causal relationship between the differences in the changes over time in patient health indicators in the two groups of patients and the PHARE-M intervention as it is often the case in complex interventions rolled out in adaptive environments. The analysis of factors associated with variations in the impact of the PHARE-M at the different trained CFCs required the adoption of instruments validated in other contexts; these could be useful for assessing the performance of other interventions in healthcare practices at CFCs in France.

## Background

Cystic fibrosis is the most common rare disease affecting the Caucasian population; it afflicts around 6500 individuals in France, 29,000 in the United States, and 11,000 in the United Kingdom. It is an autosomal recessive genetic disease caused by mutations in the cystic fibrosis transmembrane conductance regulator (*CFTR*) gene. Among all identified *CFTR* gene mutations, a list of mutations responsible for cystic fibrosis symptoms has been established and is regularly reviewed by the CFTR2 expert group [1]. Cystic fibrosis mainly affects the respiratory and digestive systems. The thick mucus in the bronchi brings about chronic inflammation and repeated infections, leading to chronic respiratory failure, the major cause of death. The majority of patients have pancreatic insufficiency and show poor nutrient absorption, resulting in an at-risk nutritional status associated with a poorer respiratory state [2]. Since the 1960s, the US Cystic Fibrosis Foundation (CFF) has identified multidisciplinary patient management at specialized centers as an essential factor in care improvement; this has led it to establish criteria for the accreditation of cystic fibrosis centers [3]. In the late 1990s, an increase in the number of adults suffering from cystic fibrosis led the CFF to clarify certain criteria for adult centers by stipulating care management by specialized physicians and a specialized team and a formalized process of transition from a pediatric center to an adult program. The accreditation process not only validates centers but also *"fosters continuous improvement efforts within care centers,"* as *"the expectation that each care center have a QI program in place was added to the accreditation and oversight process in 2004."* In the 2000s, following the publication by the US Institute of Medicine, of the report on the Quality Chasm [4], the CFF launched a benchmarking study across the US CFCs, which showed a difference of several years in the median survival age between the ten centers having the best patient outcomes and the other centers (unpublished study). This led the CFF to develop and implement a Quality Improvement Program (QIP) in the form of Learning and Leadership collaboratives [5–7] with the academic support of The Dartmouth Institute Microsystem Academy (TDIMA). A supplement in BMJ Quality and Safety has been published in May 2014 to present the success of this QI initiative [8].

In 2002, following the generalization of newborn screening in France, the French Ministry of Health designated 49 cystic fibrosis centers (CFCs) [9] and in 2006, the French National Authority for Health (HAS) published the National Diagnosis and Treatment Protocol (PNDS) in Cystic Fibrosis to establish a framework for multidisciplinary care at CFCs. The French public health insurance guarantees that every CF patient is reimbursed 100% for care and authorized drugs related to cystic fibrosis. In 2006, within the framework of the 1st National Plan for Rare Diseases, two centers of expertise for cystic fibrosis were labelled (CF-CERDs), in order to implement six priorities across the CF Network: care expertise, information systems and epidemiology, quality of care, clinical research, network organization and coordination. The Nantes/Roscoff CF-CERD, consisting of the CFCs at the two hospitals in Nantes and Roscoff as well as the transplant center in Nantes and the rehabilitation center in Roscoff, developed its action plan contributing to 5 out of the 6 priorities, covering themes such as therapeutic patient education (care expertise), quality improvement in care processes, information and communication systems, and clinical research on transplantation and in human and social science. The agreement signed by the heads of all CFCs in 2007 included a commitment to *“participate in a quality assessment and improvement program to be offered by the CF-CERDs in collaboration with the French Cystic Fibrosis Society (SFM) and the patient organizations in the next five years”*.

In 2011, the French national team at the Nantes/Roscoff CF-CERD transposed the PHARE-M quality improvement program from the US CFF QIP model (*PHARE-M: Programme Hospitalier d’Amélioration des Résultats et de l’Expertise en Mucoviscidose - Hospital Program to Improve Outcomes and Expertise in Cystic Fibrosis)*. It was launched in September 2011 with a pilot phase (2011–2012) involving seven volunteer CFCs, which underwent two external assessments, leading to certain adjustments to the initial program. This adjusted version was deployed during a regional expansion phase (2012–2013), including seven more CFCs before its national deployment [10]. The main adjustments consisted in more practical exercises during face-to-face meetings (less theoretical presentations), more on-site coaching to help the quality teams analyze their processes of care, and the designation of a PHARE-M referent in each local team to keep focused on the QI work. These 2 years are called the “experimental phase”, which involved 14 CFCs.

The two evaluations at the end of the one-year pilot phase showed the progressive adherence of the teams and improvements in care management, but a limited impact on patient health outcomes. They also highlighted that the adherence to the program mainly depended on the motivation of the multidisciplinary team (MDT), especially its lead physician. The lack of resources at some CFCs was raised to account for variations in the teams’ engagement as the level of available staff seemed to influence the extent to which the team was effectively enlisted. The participation of a patient or parent in each local quality team varied depending on the cultural context of the centers, some being used to share information with patients/parents, having a patient group in the CF center for years, others being involved in patient therapeutic education while others were acting in a more partenalistic model of care. The support received from the hospital quality department in two hospitals was emphasized as a factor that facilitated the adoption of quality tools by the teams. The recommendation of the assessor was to evaluate the impact of the program on patient outcomes by 2015.

Given the innovative nature of the QIP PHARE-M in France, the cultural differences and various organizational contexts at the CFCs, an assessment of the impact of PHARE-M at the CFCs engaged in the experimental phase was expected after 3 years to continue the enrollment in the program. Will it show favorable changes in the patient outcomes in the group of CFCs engaged in the PHARE-M compared to the other CFCs? What impact on care management can be observed in 2015? Was the period sufficient to show improvements in the two areas? In which contexts is the impact of PHARE-M observed to be the strongest? The PHARE-M Performance research project, submitted at a call for projects of the French Ministry of Health and selected for funding in December 2012, aims at providing answers to these questions.

## Methods

### A mixed methodology

The rationale of the PHARE-M Performance project is to show evidence of the performance of the PHARE-M program on patient outcomes and care management.

The study is based on a mixed methodology inspired on the one hand by epidemiology, using data from the French Cystic Fibrosis Registry, and on the other hand by the British guidelines on “Process evaluation of complex interventions” [11]:

1) *a quantitative study* to compare the changes over the 4 years in the patient health indicators of a closed cohort, using data from the French Cystic Fibrosis Patient Registry, between CFCs having benefitted from the intervention during the experimental phase and CFCs not having benefitted from the intervention up to 2015; and.

2) *a qualitative study* to analyze the contextual elements and mechanisms brought into play by the PHARE-M intervention that could account for a difference in impact among trained CFCs either on patient health indicators or on care management assessed according to the criteria of the chronic care model [12].

## Quantitative study

### Design

- observational,

- national and multi-center, and

- before/after and here/elsewhere: a comparison of patient health indicators before and after the “PHARE-M training” program at “PHARE-M Group” centers versus “Control Group” centers.

### Primary and secondary endpoints

- FEV1%.

- BMI as an absolute value and as a Z-score (standardized normal distribution of the BMI for children under 2 years of age).

For this research in particular, the value selected for these indicators is the only value appearing in the French CF Registry for a given patient and a given year. It will be analyzed by category of patients defined by age, sex, age at diagnosis, and possibly severity of disease expression, treatment, and certain social characteristics (data appearing in the Registry).

### Study population

A closed cohort was formed to identify the study population including the patients followed up at CFCs who met the following inclusion criteria according to the 2012 Registry data:

- patients seen at a CFC in 2012
- patients having two of the CF-causing mutations of the CFTR2 list published on Feb 2012
- patients not having received a transplant in 2012

A patient left the cohort if he or she no longer met the inclusion criteria after the annual data were updated in the Registry (2013, 2014, and 2015), i.e.: if he or she was a carrier of a mutation excluded from the CFTR2 list updated on 13/08/2015 [1]; if he or she was followed up at a CFC engaged in the PHARE-M in 2014 or 2015; if he or she changed CFC in the course of the study and in doing so, changed CFC group; if he or she received a transplant between 2013 and 2015 (data up to the transplantation were taken into account), or if the patient died between 2013 and 2015 (data up to the death were taken into account).

The cohort was divided into two groups: the “PHARE-M Group” and the “Control Group”:

- The “PHARE-M Group” consisted of the patients followed up at one of the 14 CFCs trained in the PHARE-M in the experimental phase (1309 patients).
- The “Control Group” consisted of the patients followed up at the CFCs not having benefitted from the intervention in the same period of time (2490 patients).

### Pairing of the two “PHARE-M” and “control” groups

A preliminary analysis of the cohort formed from the 2012 Registry data showed significant differences between the two groups of patients, before the PHARE-M intervention, in terms of: 1) distribution by age, 2) distribution by age at diagnosis, and 3) distribution by FEV1% value (see Table 1).

**Table 1:** Distribution by age, age at diagnosis and FEV1% of the 2012 study population between the two groups of the study cohort before pairing

Comparison of the two groups	PHARE-M (*N* = 1051)			Control (*N* = 1962)		
Comparison of Ages	Avg.	Med.	Max.	Avg.	Med.	Max.
Age of patients (years)	15.0	13.0	62	18.0	17.0	74
Age at diagnosis (years)	2.0	0.1	51	3.2	0.2	71
Comparison of FEV1%	Avg.	LLM	ULM	Avg.	LLM	ULM
FEV1%	83	81,55	84,45	75,48	74,33	76,64

Consequently, a 1:1 pairing of the patients from the Control Group was decided in an attempt to eliminate certain confounding factors that could be attributed to the type and size of the CFC to which the patient was assigned: each “PHARE-M patient” was associated with a “control patient” followed up at a center of the same type (pediatric, adult, or mixed) caring for a total number of patients belonging to the same interval ([1;50], [51;100], [101;150], [151;200], or [> = 200]). Reunion island CFCs were excluded from the Control Group to reduce heterogeneity in CF care. All “eligible” control patients for each patient in the PHARE-M Group were selected, and one control patient was randomly drawn from that group of eligible control patients (without replacement). The patients in the PHARE-M Group were paired in a random order.

**Fig. 1:**
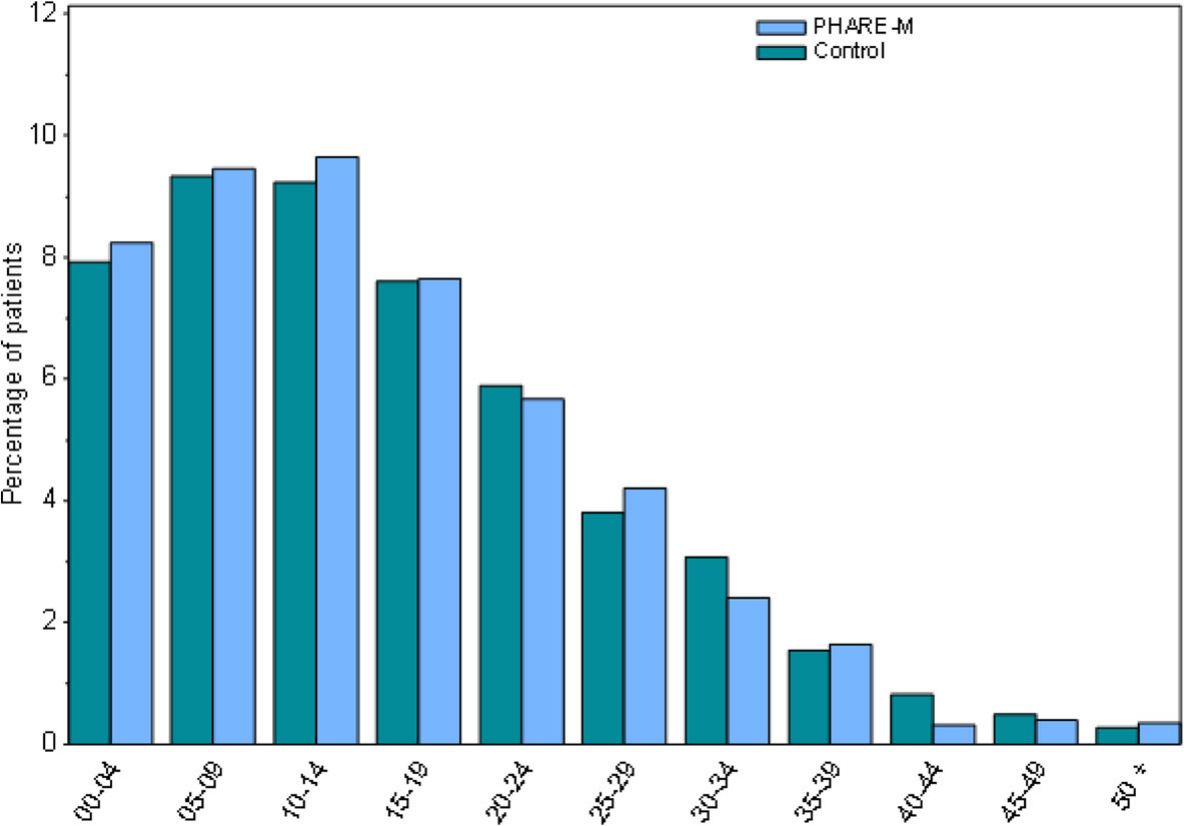
Distribution by population age between the two groups (PHARE-M and control), paired in 2012 data

At the end of the process, 1104 patients remained in each of the two paired groups. The Control Group included 20 CFCs. No paired control patients were found for 205 “PHARE-M patients”. As data are collected in the French Cystic Fibrosis Registry for all patients, exposure variables are identical in both groups. Completeness is similar: for FEV1, 20.2% and 24.5% of missing data corresponding to the children below 6 y.o., for whom this measure is not taken, and 0.6% and 3.5% for ZBMI, in the PHARE-M group and the Control group respectively. The two groups had a similar distribution by age (see Fig. 1). However, there remained a significant difference in average age at diagnosis (PHARE-M paired group: 1.9 years; control paired group: 2.5 years; *p* value: 0.0123); this could be due to the fact that newborn screening was implemented in the 1990s in Brittany, and that seven (out of the 14) CFCs in the PHARE-M Group are located in this region. Furthermore, a significant difference in FEV1% of +3.89% (*p* value = 0.0012) remained in favor of the PHARE-M patient group before the intervention (see Tables 2 and 3).

**Table 2:** Comparison between the PHARE-M Group and the paired Control Group

Comparison between PHARE-M Group and Control Group		PIIARE-M (*N* = l104)	Controles (*N* = 1104)	Patients PHARE non paired (*N* = 205)	Comparison between PIIARE-M Group and Control Group (proc TTEST)
Gender	Men *n (%)*	582 (52.72)	564 (51.09)	93 (45.37)	
	Female *n (%)*	522 (47.28)	540 (48.91)	112 (54.63)	
Age	Average	15.57	16.05	14.48	
	Std Deviation	10.73	11.00	10.51	
Age (classes)	0-04 *n (%)*	182 (16.49)	175 (15.85)	32 (15.611)	
	05-09 *n (%)*	209 (18.93)	206 (18.66)	42 (20.49)	
	10-14 *n (%)*	213 (19.29)	204 (18.48)	48 (23.41)	
	15-19 *n (%)*	169 (15.31)	168 (15.22)	38 (18.54)	
	20-24 *n (%)*	125 (11.32)	130 (11.78)	19 (927)	
	25-29 *n (%)*	93 (8.42)	84 (7.61)	10 (4.88)	
	30-34 *n (%)*	53 (4.80)	68 (6.16)	4 (1.95)	
	35-39 *n (%)*	36 (3.26)	34 (3.08)	6 (2.93)	
	40-44 *n (%)*	7 (0.63)	18 (1.63)	1 (0.49)	
	45-49 *n (%)*	9 (0.82)	11 (1.00)	4 (1.95)	
	50-54 *n (%)*	4 (0.36)	3 (0.27)	0	
	55-59 *n (%)*	4 (0.36)	2 (0.18)	0	
	60-64 *n (%)*	0	0	1 (0.49)	
	70-74 *n (%)*	0	1 (0.09)	0	
VEMS	Nmtss	223	270	49	(*p* = 0.0012 IS)
	Average	83.00	79.11	85 06	
	Std Deviation	23.96	25.81	21.92	
ZBMI	Nmiss	7	39	2	*p* = 0.5171 (NS>)
	Average	-0.17	-0.14	-0.18	
	Std Deviation	1.05	1.15	1.11	

*S* Significant; *NS* Non Significant

**Table 3:** Comparison of Age at diagnosis between PHARE-M and Control

Age at diagnosis (years)			
	Control	PHARE-M	Patients PHARE non paired
Nmiss	33	39	2
Average	2.49	1.85	2.47
Std Deviation	6.34	5.33	6.30
Comparison of Age at Diagnosis between PHARE-M and Control Groups		0.1317	*P*-value*

*Test de Wilcoxon

### Analysis of the primary endpoint between the two groups

**Fig. 2:**
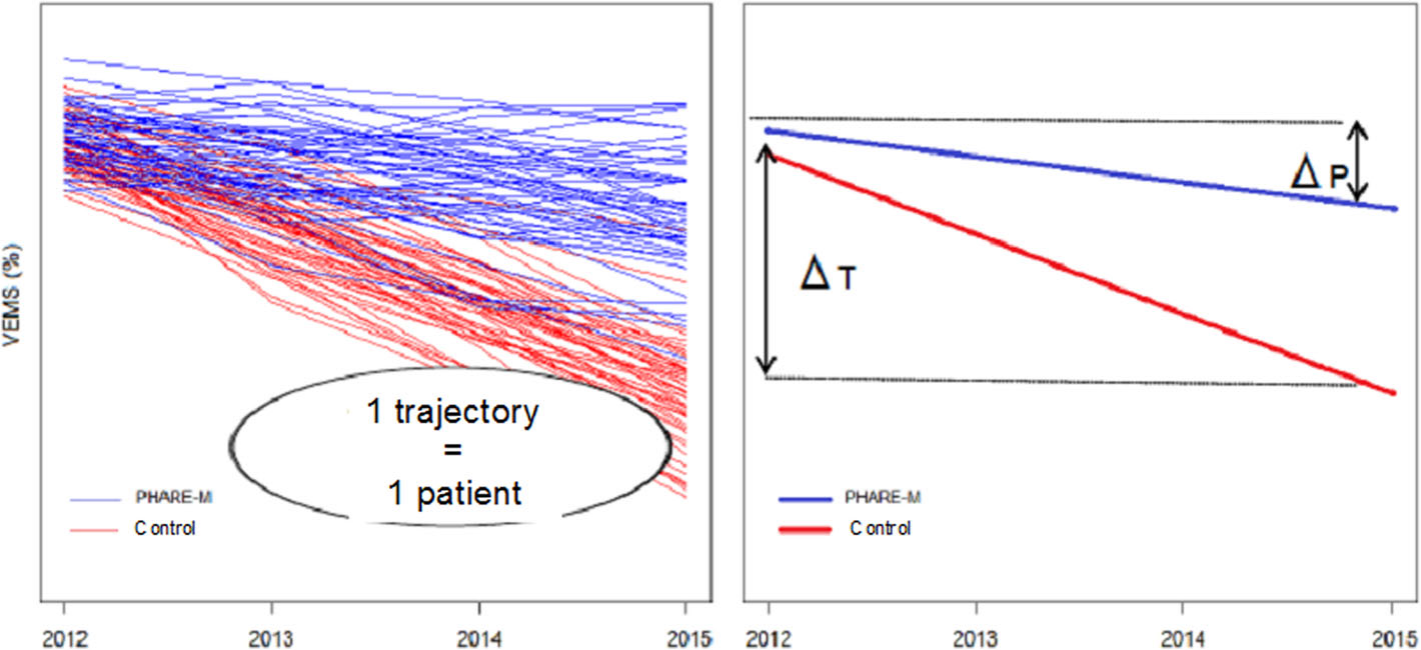
Representations of the analysis of the primary endpoint

Changes over 5 years in patient health indicators are measured for 2011 (baseline), 2012, 2013, 2014, and 2015; each patient served as his or her own control. A difference in the rate of decline is expected between the two population groups, PHARE-M and control (see Fig. 2). Changes over time in FEV1% will be modeled and compared in the two groups using a mixed model for repeated data with adjustments for potential confounding variables. Measurements for a subject *i* at time *j* is given by the following model, where *ε*_*ij*_ are the normally distributed residual components with mean zero and covariance structure Σ:

${Y}_{ij}={\beta}_0^P+{\beta}_1^P{t}_{ij}+{\varepsilon}_{ij}$ for the PHARE-M group.

${Y}_{ij}={\beta}_0^C+{\beta}_1^C{t}_{ij}+{\varepsilon}_{ij}$ for the CONTROL group

$$ \mathit{\operatorname{cov}}\left({\varepsilon}_{ij},{\varepsilon}_{ik}\right)={\sigma}_{jk} $$

The covariance structure Σ is given by the *σ*_*jk*_. It allows taking into account correlation between measurements on a same subject. Correlation is assumed to be null between subjects. The choice of a covariance structure will be data driven, but we can expect that the correlation between two measurements will only depend on the time lag between them. The most realistic covariance structure should be the so-called Toeplitz covariance matrix. A special case of the Toeplitz model is the first-order autoregressive model.

The question here is to investigate whether the two slopes are parallel or not, that is to test whether ${\beta}_1^P$ = ${\beta}_1^C$ (*H*_0_) versus ${\beta}_1^P$ ≠ ${\beta}_1^C$ (*H*_1_).

Using this model, the slopes (i.e. decline in FEV1) in the two groups will be calculated and compared. Changes over time in BMI will likewise be analyzed by comparing the changes in the two groups from 2011 to 2015, taking into account the Z-score for children under 2 years of age. The average trends will be calculated and analyzed for different patient categories (such as age, sex, age at diagnosis, severity of disease expression, treatment, and certain social characteristics in the Registry). The changes over time in indicators will be presented for the “PHARE-M Group” population by CFC for crossing with the results of the qualitative study.

### Audit of the quality of the data included in the primary endpoints’ calculation

The patient data measured by the CFCs (height, weight, and FEV1 [per L]) for 2012 and 2013 underwent an on-site quality audit at the 14 CFCs in the PHARE-M Group. It was the first on-site audit ever performed to establish the quality of these indicators. The objective was not to comprehensively audit all data for the patients included in the study. Rather, the objective was to comprehensively identify the different causes of error due to failures in the processes of measuring and/or selecting the values transmitted to the Registry in order to identify avenues for improvement of the quality of the data in the Registry. The sample of patients whose data were audited thus had to reflect the distribution by age range of the patients at each CFC (20 records/CFC) in order to cover the different measurement procedures defined by international benchmarks [13–15] and the data selection rules defined by the French Patient Registry Steering Committee, and to offer every opportunity to reach saturation of the various causes of error [16]. They will be taken into account in the interpretation of the results of the quantitative study.

## Qualitative study

### Design

**Fig. 3:**
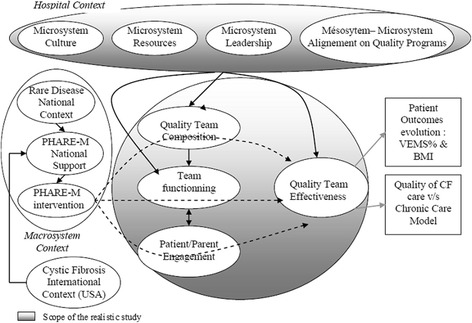
Modeling of the intervention, context, and mechanisms

The design refers to the modeling of the intervention [11] including the contextual elements and the mechanisms shown in Fig. 3.

The PHARE-M intervention consisted of establishing, training and coaching a quality team (QT) at each CFC comprising a number of professionals from the multidisciplinary CF team and 1 parent or patient from the CFC’s caseload. The members of the QT have been trained in quality methods and tools and coached in changing care processes. The PHARE-M intervention should have directly impacted the ability of the local QT to master QI methods and tools, lead changes in the care processes, and should have generated good appreciation of the utility of the QT efforts. This direct impact of PHARE-M is identified under the heading “QT effectiveness”. QT effectiveness may not only be the result of the PHARE-M intervention but may have been modulated by internal mechanisms, such as the composition of the QT (number of members and disciplines enlisted), its functioning (rigor in the QI work, decision-making, clarity of the roles…) and the parent or patient engagement. Those mechanisms are represented as impacting QT effectiveness (Table 3). Beyond the ability to master the QI methods and tools, the PHARE-M intervention was expected to have an impact on the quality of CF care delivered at the CFC. The Chronic Care Model [12] was deemed appropriate to account for quality of CF care across the 6 dimensions: existing improvement goals, multidisciplinary care, self-management support, decision support (use of evidence-based guidelines), use of information system and electronic patient record, and organization of resources in the patient’s community of life. Finally, an indirect impact of the PHARE-M intervention is expected on the trend in patient outcomes’ evolution as measured in the quantitative part of this study. Moreover, some elements in the CFC contexts, which are external to the PHARE-M intervention and preexisted to its introduction, may have had a major impact both on the adherence of the team to the QI work and on its outputs. The contextual elements that have been brought in this study include the composition of the MDT, the leadership, the patient-centeredness of care, the innovative culture of the team, and the support from the hospital quality department.

The qualitative study will test these hypotheses using a questionnaire to be self-administered, in 2015, to all members of the MDT at the 14 CFCs and to the patients/parents participating in the quality teams.

Quality of care has been defined according to the criteria of the Chronic Care Model [12]; as this model has not been popularized in France nor in cystic fibrosis, we adapted it with 47 items aimed at characterizing CF care. Table 4 presents a list of these items.

**Table 4:** Criteria for quality of CF care derived from the chronic care model

IG — Improvement Goals at the CFC	1 — There are improvement goals at the CFC
	2 — These goals, if they exist, are the subject of both indicators and an action plan at the CFC
	3 — The CFC has tools to follow up this action plan in the form of a dashboard
	4 — To your knowledge, this action plan has been discussed with management and validated
SMS — Self-Management Support - Therapeutic Patient Education	1 — To your knowledge, there is a therapeutic education program for patients at the CFC authorized by the French regional health agency (ARS)
	2 — In your opinion, the professionals at the CFC are well trained in TPE
	3 — More than 80% of the patients/parents attended at least one TPE session in the last year
	4 — The total time spent by the professionals on TPE is sufficient
	5 — There are no obstacles to implementing TPE at the CFC
	6 — The team is involved in the studies of one of the French national groups on therapeutic education via face-to-face participation or regular reporting of information
	7 — The CFC has priority objectives for developing TPE
	8 — If yes, the CFC has indicators to follow up the achievement of these priority objectives
MM — Multidisciplinary management	1 — To your knowledge, the multidisciplinary team at the CFC comprises all the disciplines recommended by the French National Diagnosis and Treatment Protocol (PNDS): specialist physician, nurse, physiotherapist, psychologist, secretary, and social worker
	2 — The number of staff in all disciplines is sufficient for the number of patients followed up
	3 — In your view, the multidisciplinary team seems stable over time (the professionals’ turnover rate is below 20% in a year)
	4 — The members of the multidisciplinary team have a great deal of expertise in managing cystic fibrosis
	5 — The multidisciplinary team meets often enough to perform a summary of the records of the patients who have come to the CFC
	6 — During these multidisciplinary meetings, the team generally reviews the records of the patients with a scheduled visit to the CFC
	7 — During these multidisciplinary meetings, the team regularly examines the patients’ educational needs and the outcomes of the educational sessions held
	8 — The scheduled consultation is genuinely multidisciplinary: the patient meets with at least the physician, the nurse, and the physiotherapist
	9 — The scheduled consultation allows the patient to meet with a professional other than the ones mentioned above, as required (dietician, psychologist, or social worker)
	10 — The scheduled consultation allows the patient to benefit at least once per year from a TPE session on a priority objective for him or her
	11 — When a patient requires it, the CFC is able to call upon a network of referent professionals in other disciplines with knowledge of cystic fibrosis (geneticist, endocrinologist, ENT, gastroenterologist, etc.)
	12 — It is possible to be managed at the CFC on a 24/7 basis
	13 — Patients who arrive at the hospital emergency department are managed in accordance with a protocol established by the CFC with the emergency department for patients suffering from cystic fibrosis
	14 — The team regularly holds a meeting to discuss its functioning and the problems at the CFC in order to improve care management
DS — Therapeutic decision support (guidelines)	1 — The team manages the availability of guidelines (nutritional, respiratory, hygienic, etc.) in a way that they are accessible to all professionals
	2 — The team has defined an internal reporting procedure to insure that care management recommendations (guidelines) updates are accessible to the team
	3 — The team systematically verifies for each patient that the latest recommendations are applied and/or offered to him or her
	4 — The team uses alerts on the population followed up to verify that the latest recommendations for care are applied to the eligible patients (e.g. glucose tolerance test alert, vaccination alert, examination alert, etc.)
	5 — The team has optimally organized the multidisciplinary consultation process (circuit, schedules, chain of professionals, cross-contamination, hazards, etc.) to deliver high quality of care.
	6 — The team has optimally organized the process of responding to telephone or email messages from the patients and families
IS — Patient information system	1 — The team uses an electronic cystic fibrosis patient record
	2 — The team has an electronic patient record system that allows it to view changes in the patient health outcomes (nutritional and respiratory outcomes) over the course of several years
	3 — The team uses the electronic patient record system during the multidisciplinary staff meetings
	4 — The team displays information from the electronic patient record during the multidisciplinary meeting (graphs of changes over time, reports from previous consultations with different professionals, etc.)
	5 — The team uses the electronic patient record system both to create alerts on applying recommendations for the patient and to compile statistics on the population followed up
	6 — The team uses the electronic patient record system to include biology results
	7 — The team uses the electronic patient record system to include imaging results
	8 — The electronic patient record system helps in selecting patients for clinical trials
	9 — The electronic patient record data are automatically transmitted with a good degree of reliability (minimal verifications, corrections, and additions) to the French Cystic Fibrosis Registry
SN — Staff in the networks in the community	1 — The CFC has organized a network of professionals in the patient community for managing care at home
	2 — The CFC organizes regular trainings for professionals in the patient community
	3 — The CFC regularly evaluates the professionals caring for CF patients in the community
	4 — The CFC assesses the health providers of devices managing CF patients
	5 — The CFC assesses the needs for home care and its distribution between professionals and carers for a balanced organization of home care
	6 — The CFC provides the patients with offers of sports activities, creative activities, and psychological support near their place of residence

QT effectiveness has been described in the studies by Lemieux-Charles [17] and Shortell [18]: it is characterized according to 27 items (see Table 5).

**Table 5:** Effectiveness of a quality team (QT)

Command of the quality process and tools	1. The teams that implement a quality process have a clear vision of the area on which to focus their improvement efforts and the expectations to be met. When you started the project, did you have such a vision?
	2. The quality teams sometimes use a method for making progress, such as a guide to follow step by step which helps them organize their work. Did your team use such a structured method?
	3. Did your team make one or more changes in its way of working?
	4. Did the team analyze data to ensure that such change(s) indeed represented an improvement?
	5. Did the team try to understand variations in the CFC processes and the reasons that could account for them (variations over time or between professionals, time of year, patient characteristics, etc.)?
	6. Does the team routinely have data allowing it to make a state of play and identify problems?
	7. Did the team have to develop a system to collect specific data (such as questionnaires, audits, interviews, or measurements) to identify problems and assess the responses provided?
	8. Did the team establish a data collection system to continue to manage quality or monitor the new processes established?
	9. Was the team able to rely on a referent professional to coordinate the meetings and work of the quality team?
	10. Was the team able to rely on a referent professional to collect and analyze data?
	1. The team was able to perform measurements to define and assess changes within the framework of tests.
Capacity to drive change	2. After testing a change, the team succeeded in discussing the outcomes observed and learning from this test.
	3. The team succeeded in analyzing the outcomes of the test to propose new changes or adjustments to be tested.
	4. During the process, the team was able to easily incorporate and adapt ideas for changes to meet the organization’s needs.
	5. The team was able to enlist sufficient knowledge and skills to drive change under good conditions.
	6. The team could find sufficient assistance in the hospital to support changes.
	7. The team could sufficiently rely on the support of the French national team to make changes at the CFC.
Effectiveness perceived by the quality team	1. The performance of the PHARE-M steering team met my expectations.
	2. I was satisfied with my experience as a member of the quality team.
	3. I believe that my participation was useful and positive for the work of the team.
	4. I would be willing to participate again on a similar team to work on quality improvement.
	5. I believe that the work of the quality team was useful for improving quality.
	6. The outcomes achieved through the work of the quality team meet the organization’s needs for improvement.
	7. It is necessary to maintain an ongoing quality improvement process to continuously improve care at the CFC.
Effectiveness perceived by the rest of the team	1. I believe that the work of the steering team was useful for improving quality at the CFC.
	2. I believe that the entire team at the CFC was enlisted and contributed to quality improvement.
	3. I believe that the outcomes achieved collectively meet the organization’s needs for improvement.
	4. I believe that it is necessary to maintain an ongoing quality improvement process to continuously improve management at the CFC.

QT Internal factors that may have modulated the QT effectiveness: QT functioning [17] is characterized by 22 items classified in 4 categories 1) the organization at work, 2) the decision-making process, 3) the shared improvement goals, and 4) the ability to communicate and get external support. Studies by L. Lemieux-Charles defined these items to analyze the impact of adopting quality improvement practices on the internal functioning of a team. We use the same items to analyze if the team’s functioning could modulate its effectiveness (see Table 6).

**Table 6:** Internal functioning of the quality team (QT)

Strictness of organization and clarity of roles	1. The leader was clear and explicit on how he or she wanted the team to work.
	2. The leader reviewed the steering team’s work and asked how we were going to go about it.
	3. The leader also requested the opinion of the other members of the team.
	4. The leader’s behavior reflected the importance he or she placed on the quality team functioning well.
	5. Our team could have been better at seeking help and securing more skills to do the work.
	6. Sometimes it seemed that we were working or going about the matter in the wrong way.
	7. Roles were so unclear that the work of different individuals seemed to overlap.
	8. The members of the team had different outlooks and experiences and came from different disciplines.
Decision- making on the QT	1. Most of the members of the team had an opportunity to participate in decision-making.
	2. We appreciated our differences, which shaped our decisions.
	3. The contribution of each member of the team was heard and taken into consideration.
	4. We examined many different ideas before making a decision.
	5. Our team possessed sufficient resources and skills and applied them well enough to work properly.
	6. Our team worked well enough to accomplish its mission satisfactorily.
Clarity of objectives	1. The members of the team were in agreement on the objectives of the project.
	2. The achievement of the objectives guided the activities of the members of the team.
	3. The members of the team did what was expected of them.
	4. The members of the team were all focused on the achievement of the same objectives.
Communication and cooperation	1. There was a great deal of cooperation between the different hospital departments.
	2. In this hospital, most departments and services have a hard time sitting down at a table and solving problems together.
	3. The people I worked with were comfortable with suggesting changes and improvements.
	4. Our team received all the information required to plan and organize its work.

The engagement of the patient/parent as characterized in Carman’s framework [19] is assessed by a list of 31 items, prepared as part of this research (see Table 7).

**Table 7:** Engagement of the patients/parents on the quality team (QT)

Information and activation of the patients/parents	1. The patients and parents are educated regularly (annually or more often) by the team about general subjects concerning cystic fibrosis care and research.
	2. The patients and parents are rather familiar with general cystic fibrosis information: research, progress made, and Registry data.
	3. The CFC team has educated the patients and parents about the PHARE-M’s importance and aim.
	4. A good relationship between the patient or parent recruited and the team is indispensable for the patient or parent to participate in the PHARE-M.
	5. The patient or parent recruited is well informed of the challenges (10 commitments) of management quality.
	6. The presence of a patient or parent on the steering team is a given and an asset.
	7. The place of a parent or patient is not on a quality team, because he or she does not have enough training or education.
	8. The place of a parent or patient is not on a quality team, because he or she already has too many personal problems to manage.
	9. The patient or parent recruited possesses the qualities to become a member of the steering team.
	10. The patient or parent recruited must have developed coping skills (see therapeutic education standard: knowing how to manage emotions and stress; solving problems, making decisions, and making choices; knowing how to communicate and being adept in relationships with others; and knowing how to put oneself in the place of others).

The context elements include: the composition of the multidisciplinary team at the beginning of the PHARE-M intervention (2011) because it might have been a limiting factor in assigning staff to the QT; the culture of the microsystem to which the QT belongs [18] i.e. the organizational culture (see Table 8) and patient centeredness and leadership style (see Table 9); the alignment of the PHARE-M QIP with the hospital quality policy as described within the framework of the European QUASER study [20] using eight open questions in an interview with a head of the hospital quality department (see Table 10).

**Table 8:** Organizational culture

*Organizational culture:*	
Research studies have defined four types of organizational culture, arising from both the organization’s external environment and internal management: a “familial” type, an “entrepreneurial” type, a “prescriptive” type, and a “productive” type. The five rubrics below describe the characteristics associated with these different types of organization. You have 100 points to distribute among the four proposals based on the degree to which they resemble your organization. For example: If the CFC resembles Description A a great deal and Description B a little, and does not resemble Description C or Description D at all, assign 70 points to Response A and the 30 remaining points to Response B.	
§1. Character	1. Organization A is very familial, like a big family. People seem to share a lot of themselves.
	2. Organization B is very dynamic and entrepreneurial. People seem to want to venture off the beaten path and take risks.
	3. Organization C is very structured and formalized. Procedures govern people’s work.
	4. Organization D is very focused on production, with the concern being that the work gets done. Individuals are not very personally involved.
§2. Management	5. Organization A’s director(s) are warm and attentive. They try to develop people’s potential and act as mentors or guides.
	6. Organization B’s director(s) take risks. They encourage people to be innovative and to try out new ideas by taking risks.
	7. Organization C’s director(s) enforce rules. They expect people to strictly apply policies and procedures.
	8. Organization D’s director(s) resemble coordinating coaches. They help people achieve the organization’s objectives.
§3. Cohesion	9. Organization A’s factors for cohesion are loyalty and tradition. Dedication to the organization is high.
	10. Organization B’s factors for cohesion are the race for innovation and development. There is a desire to be the first.
	11. Organization C’s factors for cohesion are hierarchical rules and establishment policies. Maintaining suitable functioning is important here.
	12. Organization D’s factors for cohesion are the achievement of objectives and the performance of required tasks. This vision of production is shared.
§4. Emphasis placed on...	13. Organization A emphasizes human resources. Having strong cohesion and a high sense of morale are important.
	14. Organization B emphasizes growth and acquisition of new resources. Being ready to rise to new challenges is important.
	15. Organization C emphasizes permanence and stability. Complying with rules and performing operations smoothly are important.
	16. Organization D emphasizes competition to achieve objectives. Measuring results is important.
§5. Recognition of efforts	17. Organization A recognizes all its members’ efforts equally. It is important that everybody in the pyramid, from the very top to the very bottom, is treated as equally as possible.
	18. Organization B rewards individual initiative. Those who have the most ideas and perform the most innovative actions receive the most recognition.
	19. Organization C modulates recognition based on rank. The higher your position, the more your efforts are recognized.
	20. Organization D rewards the achievement of objectives. Individuals who demonstrate leadership and thus help achieve objectives are recognized.

**Table 9:** Patient-oriented culture and leadership

Patient-oriented organization	1. Our organization works to properly identify patient needs and expectations.
	2. The professionals handle patient requests promptly.
	3. Patient complaints are analyzed to identify recurring causes and prevent problems from being replicated.
	4. The organization uses data from the patients themselves to improve services.
	5. The organization uses data regarding patient satisfaction and/or patient expectations to improve services.
Leadership at the CFC	1. The leader develops interesting/exciting opportunities for our organization.
	2. The leader proposes new and even innovative ideas to improve management services and processes.
	3. The leader drives the organization to meet patient needs and ensures management/care safety.
	4. The leader takes into account the needs of both the service and the staff during major changes within the organization.
	5. The leader builds close, positive relationships with the other departments in the hospital.
	6. The leader builds close cooperative relationships with other organizations outside the hospital.

**Table 10:** Open-ended questions to the hospital’s quality department

1. What are the priorities of the hospital’s quality department?	
2. Support for care services in quality improvement: was another quality program developed for another disease or another care service?	
3. How are patients included in the different committees and groups working to improve quality in the hospital?	
4. How is quality measured (main indicators)?	
5. What training programs in quality tools and methods are promoted by the hospital?	
6. How was the quality department informed of the PHARE-M (by whom and when)?	
7. What were the reasons for the quality department’s engagement (or non-engagement) in the PHARE-M, in support of the CFC? In the case of engagement, what resources and time were dedicated to supporting the CFC?	
8. How is the PHARE-M perceived by the quality department management in terms of coherence with hospital policy, perceived effectiveness, and other matters? If necessary, the example of another quality improvement program rolled out in the hospital can be cited.	

Focus groups with the members of each QT were conducted by the Clinical Research Assistant, designed around four open-ended questions: 1) What changes in the organization of the CFC can be attributed to the PHARE-M? 2) What difficulties were faced at the CFC? 3) What successes were achieved? and 4) What lessons from this experience after 3 to 4 years? The results of these focus groups involving the 14 CFCs will be put in perspective with the results of the survey conducted by one assessor of the pilot phase who interviewed the 7 first CFCs on the following themes: 1) PHARE-M applicability, 2) participation of patients and parents, 3) functioning and coordination, 4) perceived benefits and costs, 5) effect on the team, 6) effect on care management, and 7) recommendations for PHARE-M national deployment.

### Development of the instruments of the realistic study

The self-administered questionnaire was developed from the instruments (cited above) translated into French, and new items prepared as part of this research to characterize quality of CF care and the degree of engagement of the patients or parents. The whole questionnaire is proposed to the members of the quality teams. A limited part of the questionnaire is proposed to the members of the MDT not on the quality team. The questionnaire has been prepared from January to June 2014 with clinicians from the Nantes/Roscoff CF-CERD and experts from the Health Education and Practice Laboratory (LEPS) at the Sorbonne Paris Cité University - Paris 13 Bobigny. It has then been tested between July and September 2014 in three teams from the Nantes/Roscoff CF-CERD (pediatric, adult, and mixed) with 29 respondents from all disciplines and the patients/parents participating in the QT. As a result of these tests, the questionnaire has been slightly adapted, essentially by rewording parts of the French translation and adding free text fields (*Questionnaire available upon request to the corresponding author*).

### On-site investigations

The investigations conducted by the clinical research assistant at the 14 PHARE-M centers take place over the course of 2.5 consecutive days per CFC. The questionnaire is self-administered successively under the supervision of the clinical research associate according to a schedule established with the team at the site, with no possibility of communication or consultation among respondents. The questionnaires and responses are managed in SurveyMonkey Software and subsequently exploited using SAS and Excel Software. The focus group is conducted at the end of the visit. Each focus group is recorded using audio equipment and transcribed in writing.

### Analyses of responses and validation of the questionnaire

Responses to the items of the questionnaire are processed anonymously. Each item receives a score on a Likert scale from one to four based on the degree to which the respondent agrees or disagrees with the proposition: “Completely disagree; Disagree; Agree; Completely agree”. “No” and “Unknown” responses are assigned a score of 0. The score is reset to 100 points and can thus be totaled by theme of the questionnaire and category of respondents. An initial descriptive analysis of the responses by CFC is returned to each quality team in the month following the on-site investigation, via a web conference, in order to validate the interpretation of the scores for the different themes and identify avenues for or obstacles to continuous care quality improvement at the CFC.

A Cronbach’s alpha test will be performed on all responses collected at the centers. Since the anticipated number of respondents is around 130 people in total for the 14 teams, this test will not allow the questionnaire to be modified for use in a larger population of respondents. It mainly aims to validate the French translations of the parts of the questionnaire coming from previous studies in English and discuss the use of the parts created within this research study.

A second level of descriptive analysis will be performed by aggregating the responses (all CFCs, by professional discipline, for resource patients/parents, and for professionals) to search for potential associations between quality of care at the CFC 3 years after the PHARE-M intervention and the effectiveness of the QT and/or the engagement of parents/patients and/or contextual elements.

After the publication of the Registry report presenting the 2015 data, changes in indicators from 2011 to 2015 will be crossed with the results of the realistic part of the study, in an attempt to identify any association in relation with more favorable changes over time in patient outcomes. A “signature” set of factors associated with a maximum/minimum impact of the PHARE-M will be sought.

### Analyses of the content of the focus groups

The content of the focus groups will be exploited (coding, categorization), processed (analysis, validity), and interpreted according to the standard thematic content analysis protocol [21]. This will be done by grouping and counting within the framework developed during the pilot phase assessment.

## Discussion and conclusion

### Scope of the study and generalization

The research program aims at identifying the impact of the PHARE-M quality improvement program 3 years after the intervention at the 14 trained CFCs, situated in different organizational and cultural contexts. It uses a mixed methodology crossing the results of a quantitative analysis based on registry data and the results of a qualitative study designed in accordance with the recommendations for research on complex interventions.

The scope of the PHARE-M intervention and thus of the research concerns the management of a singular disease in a care network organized since 2002, which represents a relatively controlled scope. Therefore, the influence of contextual elements on the PHARE-M program’s impact can be analyzed independently from other confounding factors associated with different organizations for the management of various diseases or different hospital departments running diverse specialties.

Fourteen centers volunteered to engage and test the PHARE-M program; they were not randomized. Moreover, initial assessment highlighted that team motivation is a determinant of the speed of adherence to the program. This pattern of our research, focusing on an experimental phase having enlisted volunteer centers, is to be considered in interpreting the results and developing recommendations for a successful roll- out of the PHARE-M program in the national network.

Finally, the research study on the PHARE-M intervention has a study design that could be applied in the assessment of other complex interventions at healthcare settings. Hence, this research study could inform the assessment of interventions concerning the care of rare and/or chronic diseases and the instruments needed for such assessment.

### Limitations identified and initial lessons

As a result of the experimental study based on Registry data, a study population paired between two groups (intervention and control) was defined to eliminate certain confounding factors, especially factors linked to patient age distribution. Despite this pairing, significant differences remained in terms of patient age at diagnosis and primary endpoint (FEV1%) between the two groups before the intervention, in favor of the intervention group. These initial differences could have a favorable effect for the rate of decline in FEV1% in 4 years in the intervention group [22, 23]. The question is to investigate whether the slopes are parallel or not. The difference in FEV1% will be taken into account using two different intercepts in the model, one for the intervention group and one for the control group. The patients belonging to either the “PHARE-M” group or the “Control” group will be identified in the Patient Registry with respect to their group for further analysis of their health outcomes.

Moreover, on-site quality audits of the Registry data included in the calculation of the primary endpoints showed discrepancies, mainly due to the CFCs’ interpretation of the rule for selecting the values to transmit to the Registry [16]. The volume of the discrepancies identified in the data audited could be attributed to the change of the rule applied from the 2011 registry survey. This audit points out the need for a certification process to enable a larger use of this database in epidemiologic studies or for public health or pharmacovigilance purposes.

The survey conducted for the qualitative study of the multidisciplinary teams at the 14 centers should include around 130 respondents, including at most 14 patients/parents. This number of respondents might seem low for having enough statistical power in the statistical validation of the survey instruments, especially for the parts of the questionnaire developed within this research. The survey instruments could be improved within the framework of subsequent research studies aiming, for example, at comparing quality of care between centers trained in the PHARE-M and centers untrained in the program, or at making an assessment of the quality of care before/after another intervention. Therefore, this questionnaire represents an instrument that could have further uses in the network.

### Expected results in terms of quality improvement of care

If the research study enables to identify factors promoting the adoption of the PHARE-M QIP and the maximization of its impact at CFCs, attention must be paid to the contextual elements to be worked on before or in parallel with the introduction of this program at the remaining CFCs. In the United States, the CFF has conducted “Leadership Collaborative” programs to develop leadership on multidisciplinary teams. The availability of the MDTs staff at the European standards for the number of patients followed could also represent a pre-requisite for their participation in the PHARE-M. The quality of care assessed after 3 years within the CFCs trained to PHARE-M might also enable to identify new avenues for improvement, including some beyond the scope of the clinical microsystem such as the Information System or the generalization of Guidelines.
